# Embedded Fiber Bragg Grating Sensors for Monitoring Temperature and Thermo-Elastic Deformations in a Carbon Fiber Optical Bench

**DOI:** 10.3390/s23146499

**Published:** 2023-07-18

**Authors:** Ana Fernández-Medina, Malte Frövel, Raquel López Heredero, Tomás Belenguer, Antonia de la Torre, Carolina Moravec, Ricardo San Julián, Alejandro Gonzalo, María Cebollero, Alberto Álvarez-Herrero

**Affiliations:** National Institute for Aerospace Technology (INTA), Carretera de Ajalvir km 4, 28850 Torrejón de Ardoz, Spain

**Keywords:** optical bench, Carbon Fiber Reinforce Polymer, fiber bragg gratings, multiplexing, temperature measurement, strain measurement, temperature compensation

## Abstract

A composite optical bench made up of Carbon Fiber Reinforced Polymer (CFRP) skin and aluminum honeycomb has been developed for the Tunable Magnetograph instrument (TuMag) for the SUNRISE III mission within the NASA Long Duration Balloon Program. This optical bench has been designed to meet lightweight and low sensitivity to thermal gradient requirements, resulting in a low Coefficient of Thermal Expansion (CTE). In addition to the flight model, a breadboard model identical to the flight one has been manufactured, including embedded fiber Bragg temperature and strain sensors. The aim of this is to explore if the use of distributed fiber Bragg gratings (FBGs) can provide valuable information for strain and temperature mapping of an optical instrument on board a space mission during its operation as well as its on-ground testing. Furthermore, surface-mounted strain FBG sensors and thermocouples have been installed in the optical bench for intercomparison purposes. This paper presents the results obtained from a thermal vacuum test consisting of three thermal cycles with stabilization steps at 100 °C, 60 °C, 20 °C and −20 °C. Experimental results provide information about how FBG embedded temperature sensors can provide a proper and quick response to the temperature changes of the optical bench and that embedded FBG strain sensors are able to measure micro-deformation induced in a close-to-zero CTE optical bench.

## 1. Introduction

Since Kenneth O. Hill et al. reported the first work on photosensitivity in Ge-doped core optical fibers [1,2] in 1974, a large number of publications describing the use of FBGs in communications [3,4,5,6] and sensing [7,8,9] have been published. The current work is focused on temperature and thermo-elastic deformation monitoring of the TuMag optical bench breadboard using FBG sensing technology. TuMag [10] is an imaging spectropolarimeter, one of the three post-focal instruments onboard the SUNRISE III mission [11] within the NASA Long Duration Balloon Program. This mission is dedicated to the investigation of the key processes governing the physics of the magnetic field and the convective plasma flows in the solar lower atmosphere.

The TuMag optical bench has been designed in CFRP to meet the following design drivers: lightness and high dimensional stability in the presence of thermal gradients. In the breadboard model, some FBGs have been embedded in the top skin of the optical bench panel. Due to their small size, the FBG sensors present good compatibility with CFRP structures. The optical fiber, in which the FBGs are inscribed, can be routed inside the CFRP skin in such a way that it is imperceptible to the eye and touch. Furthermore, the use of embedded FBGs avoids the need for wires and adhesives on the surface that could interfere with the optical experiments. FBGs provide thermo-elastic strain monitoring and have advantages over electrical gauges: electromagnetic interference immunity, no distance-dependent wires, and no need for return fibers. The temperature sensors based on FBGs are able to work in harsh environments and at cryogenic temperatures [12], as well as at very high temperatures around 1000 °C in the case of FBGs inscribed in sapphire fibers [13,14,15]. Although the use of FBGs embedded in CFRP is well known in the literature [16,17], this is the first application where FBGs have been embedded into an optical bench to our knowledge. Temperature sensors based on FBGs may play an important role during the cruise phase of optical instruments, monitoring temperature changes in both the optical bench and the optomechanical parts. Moreover, FBG-embedded strain sensors can measure quasi-static loads due to temperature changes as well as the deformation of structures in the field of adaptive optics, taking advantage of their condition as a non-invasive measuring technique.

This work has been divided into several sections. The next section begins with a brief introduction to the theoretical fundamentals of temperature and strain measurement using embedded FBGs and describes the optical bench and the design of the sensors embedded in it. This section ends with a brief presentation of the facilities in which the test was carried out and a description of the method to be used for the analysis of the results. This method is explained in detail in Appendix A. In the Section 3, the raw data are presented. These data are treated using the methods described in the Section 2, for further discussion in Section 4. The article concludes with a summary of the achievements and conclusions drawn from the data.

## 2. Materials and Methods

### 2.1. Fundamentals

A FBG consists of a periodic modulation of the index of refraction of the core of an optical fiber [1,2]. Such a modulation can be obtained through holographic techniques [18], phase mask [19] or point-by-point techniques [20]. In the case of a modulation perpendicular to the axis of the fiber, each single core modulation reflects light backwards. By means of coupled-mode analysis, the Bragg condition can be obtained (Equation (1)) [21]:

(1) $$\lambda =2n_{eff}\;\Lambda$$

where Λ is the grating period and $n_{eff}$ is the effective index of refraction of the fiber core.

#### 2.1.1. Temperature and Strain Sensitivity of FBGs

The peak wavelength mentioned above is sensitive to both elongation and temperature changes. On the basis of Equation (1), considering changes in temperature and deformation, the Bragg wavelength changes are given by [21];

(2) $$∆\lambda =2\left(\Delta \frac{\delta n_{eff}}{\delta l}+n_{eff}\frac{\delta \Delta }{\delta l}\right)\Delta l+2\left(\Lambda \frac{\delta n_{eff}}{\delta T}+n_{eff}\frac{\delta \Lambda }{\delta T}\right)\Delta T$$

From Equation (2), it is possible to obtain Equation (3), which provides the mathematical expression for the variation of the FBG peak wavelength with respect to temperature and strain:

(3) $$\frac{∆\lambda }{\lambda_{o}}=k\varepsilon +\alpha_{\delta }\Delta T$$

where Δλ is the wavelength shift; $\lambda_{0}$ is the base wavelength; ε is the strain; ΔT is the temperature change and $\alpha_{\delta }\;$is the thermo-optic coefficient of the optical fiber;

(4) $$\alpha_{\delta }=\frac{\delta n/n}{\delta T}$$

Finally, $k=1-p_{e}$ is the gage factor, and $p_{e}\;$is the strain-optic constant defined by Equation (5).

(5) $$p_{e}=\frac{n_{eff}^{2}}{2}\left[p_{12}-\upsilon \left(p_{11}+p_{12}\right)\right]$$

with $p_{11}$ and $p_{12}$ components of the strain-optic tensor and υ is the Poisson’s ratio [22] of the optical fiber.

It has to be noted that in Equation (3), the strain is defined by:

(6) $$\varepsilon =\varepsilon_{m}+\varepsilon_{T}$$

where $\varepsilon_{m}$ is the mechanically induced strain and $\varepsilon_{T}=\alpha_{glass}\Delta T$ is the temperature-induced strain, where $\alpha_{glass}$ is the coefficient of thermal expansion per K of the optical fiber. In this case, the optical fiber is made of glass. Equation (6) highlights the cross-sensitivity between strain and temperature. The temperature that induces strain depends on the coefficient of expansion, which, in turn, depends on the temperature.

Combining Equations (3), (5) and (6), Equation (7) can be obtained, which describes the wavelength shift for a FBG due to temperature changes and strain:

(7) $$\frac{∆\lambda }{\lambda_{o}}=k\left(\varepsilon_{m}+\alpha_{glass}\Delta T\right)+\alpha_{\delta }\Delta T$$

In the particular case of an FBG sensor that is not subjected to any mechanical load, there are no mechanically induced deformations in the FBG, and the wavelength shift does not depend on mechanical strain, so that Equation (7) can be expressed as:

(8) $$\frac{∆\lambda }{\lambda_{o}}=(k\alpha_{glass}+\alpha_{\delta })\Delta T$$

For practical purposes, Equation (8) is the mathematical representation of an FBG sensor working as a temperature sensor.

#### 2.1.2. Strain Measurement with Temperature Compensation

The literature refers to many techniques for performing temperature compensation for strain measurements [9,21,23]. In the present work, a method was used that utilizes two identical FBGs, inscribed in the same type of optical fiber and with the same grating period (Λ). One of the FBGs acts as a strain sensor; this sensor must be temperature-compensated. The strain sensor thus plays the role of the strain-measuring sensor. The other FBG sensor, the compensation sensor, must be located close to the first one. Therefore, it can be assumed that both sensors are subjected to the same temperature variation. The temperature compensation sensor must be isolated from any external mechanical load. If we consider Equation (7) to represent the strain measuring sensor and Equation (8) for the temperature compensation sensor, then by combining both equations, the mathematical expression of temperature-compensated strain${\;(\varepsilon }_{m})$ can be obtained:

(9) $$\varepsilon_{m}=\frac{1}{k}\left(\frac{∆\lambda_{m}}{\lambda_{om}}-\frac{∆\lambda_{c}}{\lambda_{oc}}\right)$$

where $∆\lambda_{m}$ and $∆\lambda_{c}$ are the wavelength shifts of measuring and compensation FBGs, respectively; $\lambda_{0m}$ and $\lambda_{0c}$ are the base or reference wavelengths for measuring and compensation sensors and k is the calibration factor that is characteristic of the optical bench structure. The factor *k* is also strain and temperature dependent [22,24].

### 2.2. Optical Bench BB Design and Description

The optical bench is a sandwich panel consisting of a core of 40 mm thick AL5056 Honeycomb (cell size of ¼ inch, nominal foil gauge of 0.0015 inch and density 3.4 PFC) and skins of CFRP made of Torayca Prepeg M40J-MTM44-1 with high modulus fiber and epoxy matrix. The stacking sequence of the skin is [+45/90/−45/0]_2s_ to obtain quasi-isotropic properties in-plane directions. This CFRP sandwich panel design ensures a lightweight bench with low thermal-mechanical deformations, high stiffness and near-zero CTE. A set of aluminum inserts is used for attaching all the optical elements and assemblies of the TuMag instrument. The stability of the distance of the inserts in the presence of thermal gradients and the flatness of the optical bench are two of the most important design drivers due to their impact on the optical performance of the TuMag or any other optical instrument; the location tolerance for drill holes is ±0.1 mm and for pin holes is ±0.02 mm. The required optical bench flatness for the current application is ±0.8 mm for the whole bench, which is obtained by machining and grinding the insert surface. Considering the large dimensions of the optical bench (846 mm long and 496 mm wide), these requirements are very restrictive.

The upper skin of the optical bench contains eight surface-mounted fiber connectors. The connector housings have been manufactured by Epsilon Optics Ltd. (Fordingbridge, UK) in a 3D-printed thermoplastic based on Somos^®^ PerFORM material by DSM Company (Heerlen, The Netherlands). The performance of these connectors has been evaluated after panel curing and during thermal testing.

The following figure presents a diagram of the installation of the FBG sensors on the optical bench.

### 2.3. Sensors Design

All the FBG embedded sensors have been installed in the upper skin of the TuMag optical bench breadboard. The FBGs are drawn tower gratings of 4 mm length, inscribed using the femtosecond technique and manufactured in polyimide-coated low-attenuation fiber by FBGs Technologies GmbH (Jena, Germany).

**Figure 1:**
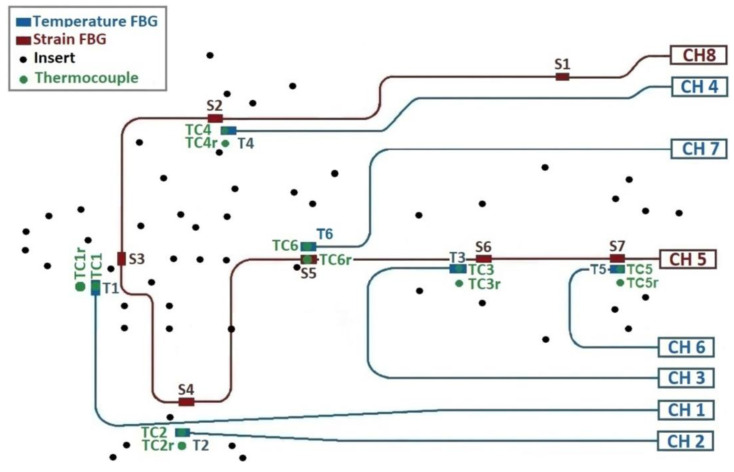
Diagrammatic sketch of the optical bench: The FBG interrogation channels are labeled CH1 to CH8. The FBG strain sensor array (S1–S7) is connected to CH8 and is shown in red. In addition, a surface-mounted strain sensor array is connected to CH5. The FBG temperature sensors T1, T2, T3, T4, T5 and T6 are shown in blue. The thermocouples, main (TC1 to TC6) and redundant (TC1r to TC6r), are presented in green. The aluminum inserts are represented by black circles.

Figure 1 shows a sketch of the allocation of all the sensors installed in the optical bench during the thermal test, as well as the location of the inserts in the optical bench and the corresponding channels of the optical fiber sensor in the interrogation equipment.

Next, the description of both the temperature and strain sensors is presented in detail.

#### 2.3.1. Temperature Sensors

There are six FBG-embedded temperature sensors in six independent optical fibers, labeled T1, T2, T3, T4, T5 and T6. The expected temperature sensitivity is 10 pm/K, and the wavelength peak at room conditions is presented in Table 1. Additionally, there are two intercomparison thermocouples for each FBG temperature sensor: a main (TC1, TC2, TC3, TC4, TC5 and TC6) and a redundant (TC1r, TC2r, TC3r, TC4r, TC5r and TC6r) (see Figure 1).

**Figure 2:**
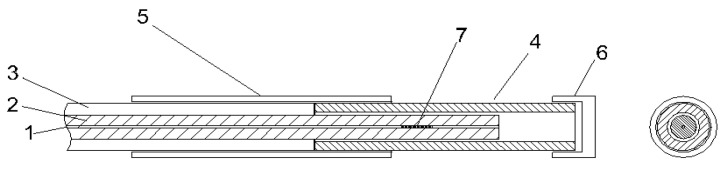
FBG temperature sensor detail [25]: (6) Tube lid; (5) protection tubing; (3) Optical fiber; (7) FBG sensor; (1) Optical fiber core; (2) Optical fiber jacket; (4) Stainless-steel tube.

In order to meet the compensation sensor requirements outlined in Section 2.1.2, the temperature sensors must be able to move freely and must not be subjected to any external mechanical load. In most experimental setups, it is enough to simply glue the sensor to the surface at one end, leaving the other end free. The situation for embedded temperature sensors is more complex since they are stacked within the structure. At INTA, a technique has been developed for embedding temperature sensors in composites with minimal structural impact. Figure 2 presents the design for a temperature sensor based on an FBG. This fiber is inserted into a stainless-steel tube with an inner diameter of 150 microns, which is slightly bigger than the 125 microns of the uncoated fiber. The fiber and the tube are then glued together with a protective tube so that the uncoated end of the fiber can move freely.

#### 2.3.2. Strain Sensors

The embedded strain FBG sensor consists of an array of seven FBGs with wavelengths between 1520 nm and 1550 nm, spaced at 5 nm.

Figure 1 presents the routing of the strain array. The location of each FBG is determined by the thermal test objectives of the optical bench. The sensors from S2, S3, S4, S5, S6 and S7 are located in several zones with a high density of inserts underneath the main subsystem that constitutes the TuMag instrument. The S1 sensor is placed in a zone free of inserts. Additionally, for redundancy and intercomparison, a surface-mounted sensor array has been glued to the top skin of the optical bench, using EPO-TEK 353ND epoxy resin. The surface-mounted multiplexed strain sensor is the same as the embedded one and has been implemented for redundancy and intercomparison purposes.

During the process of installing the inserts, the embedded FBGs S5, S6 and S7 were lost due to an issue with the machining of the optical bench. This problem also caused a loss of reflectivity in the sensors S1, S2, S3 and S4.

### 2.4. Thermal Vacuum Test Description

**Figure 3:**
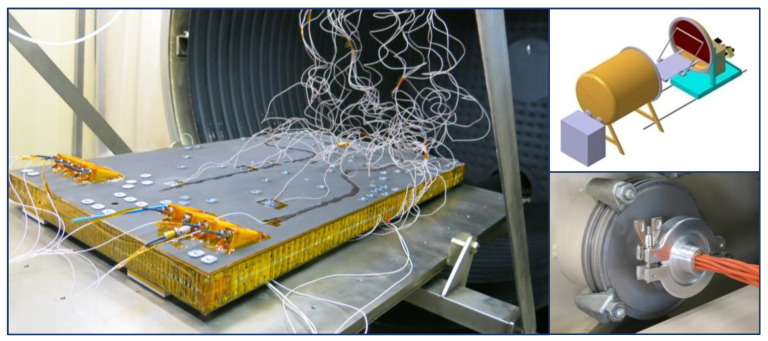
TuMag optical bench breadboard thermal vacuum test: The figure presents the CFRP optical bench in its test configuration; the CAD model of the thermal vacuum chamber and the in-house manufactured vacuum compatible fiber optic feedthrough.

The thermal testing of the optical bench has been carried out at INTA in a thermal vacuum chamber. The thermal chamber was equipped with a temperature-controlled shroud and a baseplate capable of testing in the range of −165 °C to 165 °C and down to 10^−6^ mbar ambient pressure (Figure 3).

The test consisted of three thermal cycles with four stabilization temperatures each. It was performed under vacuum conditions below 10^−5^ mbar. The test began at 100 °C in order to ensure the evaporation of ambient humidity from the optical bench. The stabilization points were 100 °C, 60 °C, 20 °C and −20 °C.

The FBG sensors were read by a Hyperion Si255 by LUNA (Roanoke, Virginia, USA) optical interrogator with eight measuring channels. This interrogator features 1 pm wavelength stability and 1 pm repeatability according to supplier specifications. An in-house-manufactured vacuum-compatible fiber optic feedthrough was used to connect the embedded FBGs to the interrogator. Six channels were used for the FBG temperature sensors and the other two for the FBG strain sensors.

The optical bench was also equipped with two heaters for a thermal conductivity test. The temperature effects of the heaters were observed by the thermocouples and the embedded FBGs, as shown below.

### 2.5. Peak Wavelength and Spectral Response Detection

Data detection for FBG was carried out in the range of 1460 nm to 1620 nm. Peak wavelength detection was performed using two different, complementary methods. The peak wavelength of each FBG was monitored at 100 Hz using the LUNA Peak Detection application of the Enlight 1.18.8.0 Analysis Software. Additionally, the spectral response of the FBGs of each channel was recorded every thirty minutes with a step resolution of 8 pm. Information regarding the location of the FBG peak could also be extracted from the spectral data. A tailored MATLAB algorithm was used to extract the peak, even for peaks of 3 or 4 dBm. The method used to extract the peak wavelength from the spectrum data is explained in Appendix A.

## 3. Results

### 3.1. Temperature Sensors

**Figure 4:**
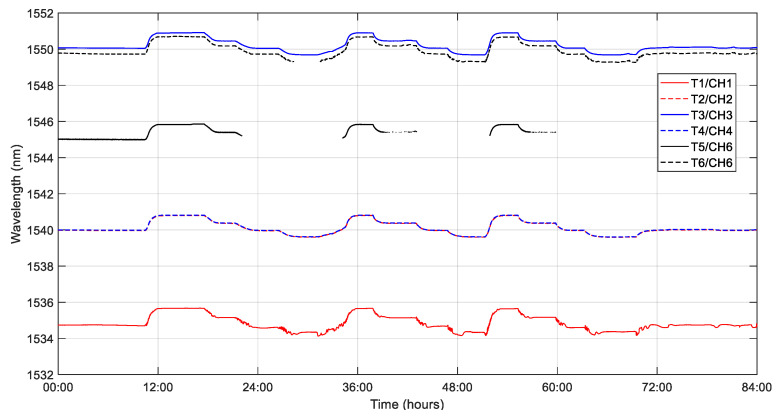
Temporal evolution of the peak wavelength for the FBG embedded temperature sensors T1, T2, T3, T4, T5 and T6 from the start to the end of the test.

In this section, the raw temperature data are presented. Figure 4 presents the temporal evolution of the peak wavelength recorded by the LUNA Peak Detection application for sensors T1, T2, T3, T5 and T6. It is worth noting that, although there are missing data points in the time evolution of T5, the peak wavelength can be recovered in the stabilization stages by the tailored MATLAB algorithm mentioned above. It is also interesting to observe that the data for sensors T1 and T6 present noisy time evolution. These results will be discussed in Section 4.1.

**Figure 5:**
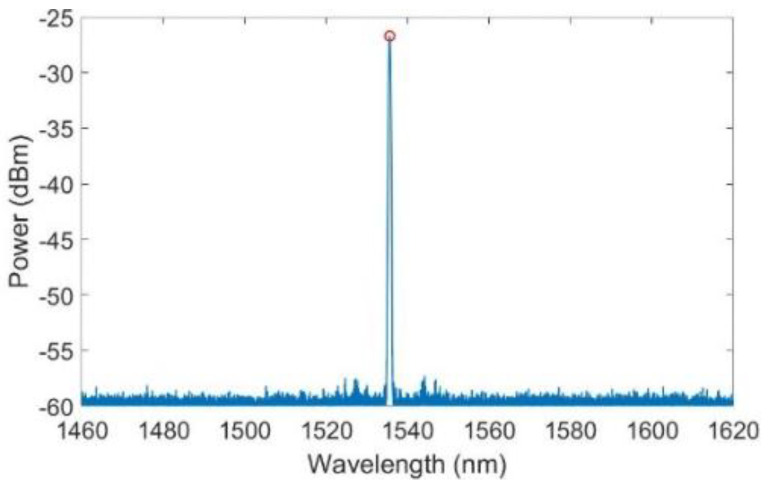
The T1 sensor spectrum at 100 °C obtained by the Si255 LUNA Hyperion optical interrogator.

We can also work with spectral data to obtain the peak wavelength of each sensor in the stabilization stages. An example of such data can be found in Figure 5. According to the methodology outlined in Section 2.5, once the peak wavelength is obtained, the temperature data can be retrieved by using the calibration curve of the sensor.

### 3.2. Strain Sensors

**Figure 6:**
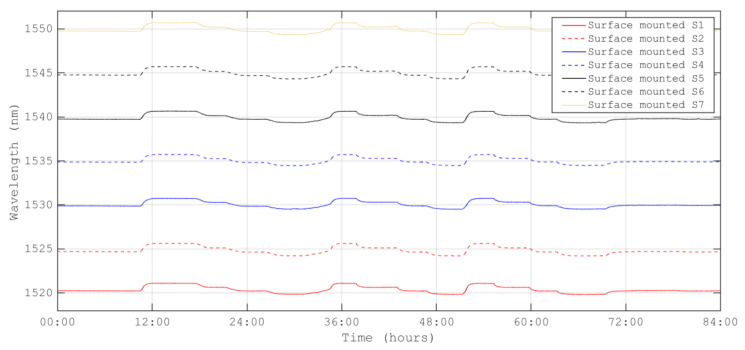
Peak wavelength temporal evolution for surface-mounted FBG strain sensors.

**Figure 7:**
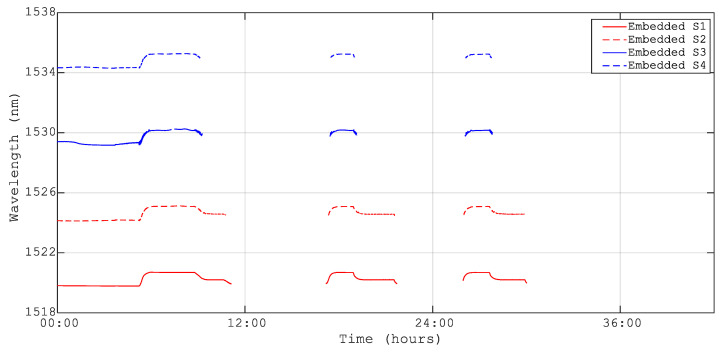
Peak wavelength temporal evolution for the embedded FBG strain sensors.

As for temperature sensors, the peak wavelength of the strain sensor data was recorded in two ways: from the peak detection application of the LUNA interrogator and from the FBG spectrum in the stabilization stages of the thermal test. Figure 6 and Figure 7 present the data for embedded and surface-mounted strain sensors, respectively.

For the embedded sensors, some data were missing due to an issue during manufacturing when the fiber was cut by the drilling tool, resulting in a Fabry–Perot effect that reduced the reflectivity of the strain sensor in the line. Additionally, the issue with the surface connector contributed to signal loss at low temperatures. Missing data have been retrieved using a tailored MATLAB algorithm. The strain results have been calculated using Equation (9) from the data in Figure 6 and Figure 7, with a gauge constant of k = 0.76 based on previous experience with the quasi-isotropic carbon/epoxy composite material M21/T800 [24].

## 4. Discussion

### 4.1. Temperature Monitoring

According to Equation (8), the slope of the linear fit of the temperature data (Table 2) is the experimentally calculated sensitivity of the FBG temperature sensor. One of the requirements for obtaining a properly working FBG sensor is that the optical fiber moves freely inside its stainless-steel housing (see Figure 2). From now on, “free slope” will refer to the sensitivity of the sensor when free and in air, which is approximately 10 pm/K, and “embedded slope” will refer to the sensitivity of the embedded sensor measured during the thermal test (Table 2). Comparing the free slope with the embedded slope allows us to determine the freedom of movement of the fiber in its stainless-steel tube after manufacturing.

Table 2 presents the sensitivity measured for each temperature sensor between 100 °C and −20 °C during the thermal test. The embedded slopes of T1, T5 and T6 are far from the value of 10 pm/K of the free slope, and the statistical error of sensitivity is higher than for T2, T3 and T4. This may suggest that T1, T5 and T6 are obstructed in their tubes, have lost their freedom of movement, and will be disregarded in the calculation of the temperature-compensated strain.

The sensor T1 is placed perpendicular to the surrounding carbon fibers, and therefore, the stainless-steel tube may be deformed by the surrounding carbon fibers. The deformation of the tube could block the free movement of the FBG inside the tube. To solve this problem, two possible solutions could be considered for future developments: The first is to always place the FBG temperature sensor always parallel to the surrounding carbon fibers, and the second could be to increase the stiffness of the FBG temperature sensor tube.

From the previous paragraph, it can be concluded that the linear fit of the peak wavelength data can be a good tool to evaluate the quality of the FBG temperature sensors. For small temperature ranges, close to room temperature, the inverse linear fit slope can be a good approximation in order to translate wavelength peak data into temperature data. However, since the fiber thermo-optical coefficient and the coefficient of thermal expansion are not constant with temperature [26], the inverse linear fit cannot be used to perform a reliable temperature calibration of the peak wavelength data for wide temperature ranges and cryogenic temperatures. For the case under study, the peak wavelength has been fitted using high-order polynomials, and it has been experimentally observed that the cubic degree is the lowest order that presents a good fit between the temperature data measured by thermocouples and by the FBG temperature sensors.

**Figure 8:**
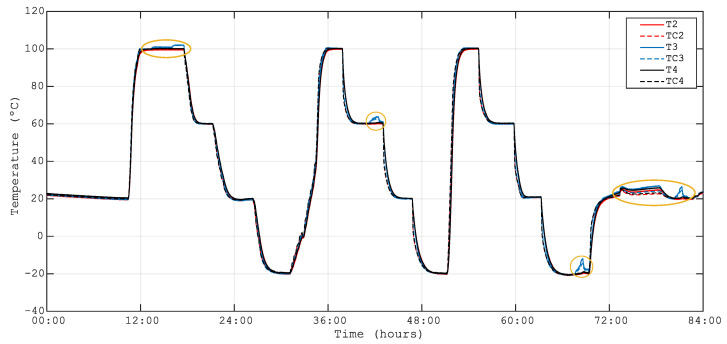
Temperature evolution from 100 °C to −20 °C; T2, T3 and T4 are FBG temperature sensors, and TC2, TC3 and TC4 are thermocouples for intercomparison. The data encircles in orange are not considered in the data analysis of the current study.

Figure 8 presents the time evolution of the temperature measured by thermocouples TC2, TC3 and TC4 located near the FBG temperature sensors T2, T3 and T4, respectively. The temperature measurement of the FBG temperature sensors T2, T3 and T4 is also plotted by cubic fitting with the wavelength data shown in Figure 4. From Figure 8, it can be said that there is an excellent agreement between the temperature of FBGs and thermocouples in the range from −20 °C to 100 °C. Therefore, a third-order polynomial calibration is proposed for embedded FBG temperature sensors in the studied range.

The orange ellipses shown in Figure 8 correspond to the time at which the heaters used in the parallel test described in Section 2.4 were switched on. The data from these regions have been discarded for the present study since the heaters break the thermal stability of the optical bench.

It is interesting to note that, as shown in Figure 8, the temperature of the optical bench, measured by the FBG temperature sensors T1, T2 and T3, has been considerably homogeneous during the stabilization stages of the test.

**Figure 9:**
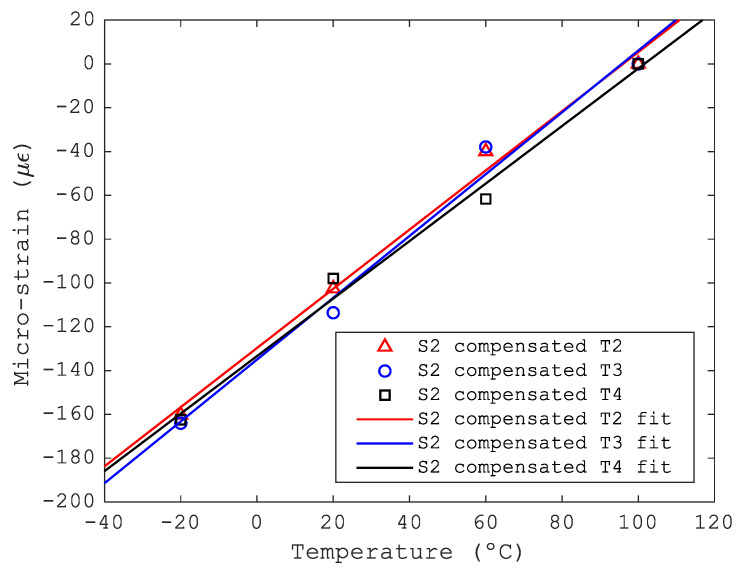
Measured micro-strain S2. Comparison between temperature compensation using T2, T3 and T4.

As shown in Figure 8, the temperature during the stabilization stages was extremely homogeneous on the top skin of the bench. Figure 9 presents the FBG surface-mounted sensor S2 micro-strain, calculated as shown in Section 2.1.2, and compensated by three different FBG temperature sensors T2, T3 and T4 placed in different locations. The magnitude of the micro-strain obtained with different compensation sensors was similar for sensor S2 (see Figure 9). To improve the information about the surface-mounted sensor S2, the variation rate of micro-strain with temperature has been calculated by linear fitting. The results are presented in Table 3. The rate of change of micro-strain with temperature obtained for compensation sensors T2, T3 and T4 differed by 4%. The same exercise has been carried out for all the surface-mounted strain sensors, and the results are presented in Table 4.

Although Figure 9 and Table 3 present excellent agreement between the micro-strain obtained with three different temperature sensors, column 4 of Table 4 shows that the agreement is not as good for S3 and S4. From the analysis of the data in Table 4, it can be seen that for small values of strain (see column 2 of Table 4), the difference in the rate of variation of micro-strain is very high. This could be explained because, to carry out the temperature compensation of the deformation measurements, it is necessary to have a temperature sensor as close as possible to the deformation sensor in question. In this case, we have used data from a temperature sensor relatively far from the deformation sensor. If we consider that the bench is in a stable and homogeneous temperature regime, this approximation would be valid. However, if the deformation occurring is very small, then the temperature compensation of the deformation sensor obtained by a very distant sensor may not be valid, and this is why it presents such a large relative difference. In these cases, it can be concluded that for values below about 50 με, the difference in the micro-strain variation rate is too high to allow compensation with sensors located at large distances. For micro-strain values above 50 με, however, there seems to be a good agreement between the micro-strain rates when temperature compensation is carried out with a temperature sensor far away from the strain FBG sensor, as long as we consider a homogeneous and temperature-stabilized optical bench.

Table 4’s second column presents the average value of the micro-strain measured by the sensor in the thermal range from 100 °C to −20 °C, along with its standard deviation. The third column presents the micro-strain rate of change with temperature obtained by linear fit and its statistical error. The fourth column presents the maximum difference, as a percentage of the total, found in the variation rate when temperature compensation has been carried out with either sensor T2 or T4.

The data in Table 4 also informs us about the mechanical behavior of the optical bench during the thermal range from 100 °C to −20 °C. Most of the sensors measured local contraction except for sensor S3, which measured small local dilatation. The maximum elongation obtained for the 846 mm long TuMag optical bench, calculated by multiplying the length of the optical bench by the maximum value of the measured micro-strain, was 0.16 mm for the measured thermal range of 120 °C. This value is in agreement with the expected value for an optical bench with a CTE close to zero.

Table 5 presents the comparison between the surface-mounted FBG sensors and the embedded FBG sensors. It is important to note that for the embedded FBG sensor S1, there are data at 20 °C, 60 °C and 100 °C and for the embedded FBG sensors S2, S3 and S5, there are data at 60 °C and 100 °C. The table presents the average micro-strain value obtained from the spectrum at the stabilization temperatures and its corresponding standard deviation. The difference between the micro-strain measured between the embedded and surface-mounted FBG strain sensors is also presented.

To understand the results of Table 5, it is interesting to note that the distance from the neutral flexural axis of the panel to the embedded sensors is different than for the surface-mounted sensors, which are bounded on the top of the bench. The surface-mounted FBGs are 3 mm further from the neutral axis than the embedded ones. In light of this difference, it can be said that the embedded and surface-mounted sensors are subjected to different strains, and for this reason, they measure different strain values. Such a difference, added to the experimental and computational errors, could explain the difference found between the strain values measured by surface-mounted FBG sensors and embedded FBG sensors.

According to theory [22,23], embedded FBG strain sensors are more sensitive to transverse and longitudinal strain than the surface-mounted ones. A surface-mounted FBG strain sensor needs a minimum effective bound length to transfer the strain to the fiber core. This minimum longitudinal length is about 3 mm for polyamide-coated FBGs and 10 mm for an acrylate-coated one [23]. The fiber diameter is only 0.25 mm, so the transverse strain is not transmitted to the core of the optical fiber. Consequently, surface-mounted FBGs are only sensitive to longitudinal strain. The elastic module of the adhesive is also decisive in the transfer of the strain to the fiber; the adhesive must be stiff enough to transmit the strain to the optical fiber [23]. It can be observed that the data obtained by an embedded and a surface-mounted strain FBG sensor are strain direction-dependent. In addition, adhesives can absorb moisture and/or change their physical properties in harsh environments, so a surface-mounted sensor introduces more distortion into the experimental results than embedded ones [23].

Embedded FBG strain sensors present several advantages compared to surface-mounted ones, the most important of which is improved measurement accuracy.

### 4.2. Surface-Mounted Connectors

The performance of the FBG sensors was checked in lab conditions after the optical bench insert machining, and they were found to be working properly. After the thermal test, a visual inspection was carried out, and it was observed that some of the surface-mounted connectors had cracks. It is hypothesized that the differential dilatation between the Perform^®^ polymers of the connector housing, the epoxy resin of the potting, and the stainless steel of the connector could have produced stress in the connectors, which could have been the cause of the connector fracture. Therefore, a new design of surface-mounted connectors, smaller than the previous ones and made entirely of stainless steel, is proposed.

## 5. Conclusions

A CFRP optical bench has been developed for the TuMag instrument for the SUNRISE III mission, meeting demanding requirements for the location of the inserts and very stringent constraints on dimensional stability with respect to temperature changes. Additionally, a breadboard optical bench identical to the flight model but including embedded FBG sensors to measure temperature and strain has been manufactured. The objective of the development is to provide information about the deformation and temperature of optical instruments onboard a space mission during ground testing and operation for future missions.

The design of FBG temperature sensors has been presented, showcasing a compromise solution between the miniature sensor housing diameter and the stacking of the carbon fiber in the optical bench. FBG temperature sensors have been characterized, and a third-order polynomial calibration has been proposed. Comparison of the temperature measurements by the FBG sensors and the thermocouples has yielded good agreement. The embedded FBG temperature sensors are very good candidates for temperature monitoring in CFRP optical benches, optomechanical parts, or assemblies.

The optical bench strain has been measured with embedded and surface-mounted FBG strain sensors, and the results obtained have been compared. It can be concluded that embedded strain sensors can provide better solutions to monitor optical bench strain for optical instruments than surface-mounted ones.

Implementing the improvements identified during the tests for surface-mounted connectors and embedded temperature sensors is the next step. The high maturity of the sensor design and the measuring strategy demonstrate a high potential for space applications.

## Figures and Tables

**Table 1:** FBG peak wavelength measured at 20 °C. The peak wavelength of the sensors has been selected to fit in the bandwidth of the optical interrogator, and they were designed with a nominal distance of 5 nm.

Sensor	Peak Wavelength (nm)	Standard Deviation (nm)
T1	1534.61	±0.03
T2	1539.947	±0.006
T3	1550.049	±0.007
T4	1539.970	±0.005
T5	1544.99	±0.03
T6	1549.710	±0.004

**Table 2:** Linear fitting results for the embedded temperature sensors. According to Equation (8), the slope of the fitting represents the sensitivity of the sensor. The third column of the table presents the statistical error of the slope of the linear least squares fit.

Sensor	Sensitivity (pm/K)	Statistical Error (pm/K)
T1	11	±2
T2	10.1	±0.8
T3	10.0	±0.5
T4	10.1	±0.6
T5	11	±1
T6	11	±2

**Table 3:** Variation rate of strain with temperature for the strain sensor S2. Temperature compensation has been performed with temperature sensors T2, T3 and T4 for comparison.

Compensation Sensor	Variation Rate of Strain (με/°C)	Statistical Error (με/°C)
T2	1.35	±0.03
T3	1.41	±0.04
T4	1.31	±0.06

**Table 4:** Variation rate of micro-strain with temperature changes measured by surface-mounted FBG sensors strain. The temperature compensation has been made with T3 for all the sensors.

Surface-Mounted Sensor	Strain (με/°C)	Variation Rate (με/°C)	Variation Rate Percentage Difference (με/°C)
S1	−35 ± 6	0.30 ± 0.02	20%
S2	−164 ± 5	1.41 ± 0.04	4%
S3	10 ± 11	−0.08 ± 0.04	55%
S4	−18 ± 6	0.16 ± 0.03	60%
S5	−80 ± 8	0.66 ± 0.03	9%
S6	−153 ± 8	1.20 ± 0.05	8%
S7	−103 ± 5	0.86 ± 0.03	13%

**Table 5:** Comparison between the results of embedded and surface-mounted strain sensors.

Sensor	Temperature (°C)	Surface Mounted (με)	Embedded (με)	Difference (με)
S1	100	0 ± 8	0 ± 6	0 ± 10
	60	−7 ± 3	−48 ± 12	−41 ± 15
	20	−25 ± 10	−30 ± 34	−5 ± 44
S2	100	0 ± 9	0 ± 8	0 ± 17
	60	−44 ± 10	−6 ± 45	38 ± 55
S3	100	0 ± 6	0 ± 9	0 ± 15
	60	4 ± 20	−13 ± 17	−17 ± 37
S4	100	0 ± 5	0 ± 9	0 ± 10
	60	0 ± 13	−20 ± 23	−20 ± 36

**Figure A1:**
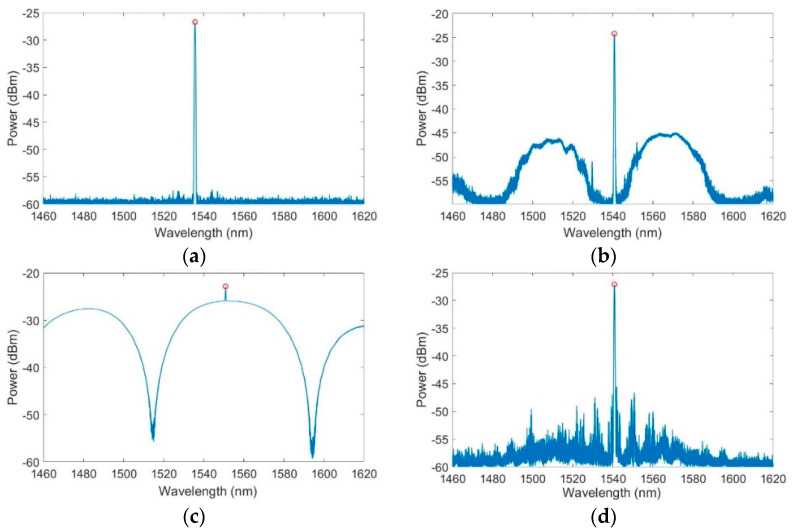

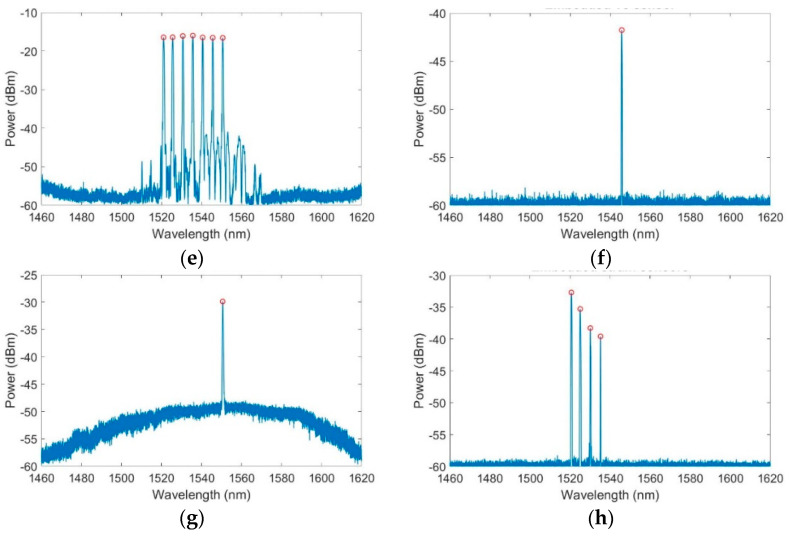
FBG’s spectral response of the eight channels. The spectral response was measured at a stable temperature of 100 °C. The spectral responses have been measured with a Si255 LUNA interrogator. (**a**) Embedded temperature sensor T1; (**b**) Embedded temperature sensor T2; (**c**) Embedded temperature sensor T3; (**d**) Embedded temperature sensor T4; (**e**) Embedded strain sensor array; (**f**) Embedded temperature sensor T5; (**g**) Embedded temperature sensor T6 and (**h**) Surface-mounted strain sensor array. The red circles in the figures correspond with the location of the peak wavelength provided by the MATLAB peak finder algorithm.

## Appendix A

The SI255 LUNA interrogator can be used to acquire the spectral response of each channel. Figure A1 presents the spectral response of each channel at 100 °C, acquired during the first cycle of the tests. It can be seen that several signals have a noisy background (Figure A1d,e,g). Figure A1b,c indicate the presence of Fabry–Perot cavities. Considering air as the filling medium, the cavity lengths can be calculated, which Table A1 presents the resultant free spectral range for both channels. The length of the cavities seems to suggest that they are located in the temperature sensors and might correspond to the space between the end of the optical fiber and the glass tube lid (see Figure 2).

**Table A1:** Fabry–Perot cavity lengths in T2 and T3 sensors at 100 °C.

Sensor	Cavity Length (μm)
T2	43.0 ± 0.7
T3	30.2 ± 0.5

From the study of the data, it can be observed that the power of the FBG peak decreases with decreasing temperature, some of them barely reaching a few dBm. Even channel 6 (embedded FBG temperature sensor T6) and channel 8 (embedded FBG strain sensor array) completely lost their peak signals at −20 °C.

The peak wavelength of some embedded sensors could not be recovered during the thermal test as the signal peak power was below the threshold considered when programming the peak detection application or the LUNA interrogator. However, with the spectral information and the tailored MATLAB algorithm, the peak for low signals could be obtained, except for channels 6 and 8 at −20 °C where the peak was unrecovered due to the total loss of the peak signal.

## References

[1] Hill K.O. (1974). Aperiodic Distributed-Parameter Waveguides for Integrated Optics. Appl. Opt..

[2] Matsuhara M., Hill K.O. (1974). Optical-Waveguide Band-Rejection Filters: Design. Appl. Opt..

[3] Ghosh C., Priye V. (2018). Dispersion Compensation in a 24 × 20 Gbps DWDM System by Cascaded Chirped FBGs. Optik.

[4] Goh C.S., Set S.Y., Kikuchi K. (2002). Widely Tunable Optical Filters Based on Fiber Bragg Gratings. IEEE Photon. Technol. Lett..

[5] Sayed A.F., Mustafa F.M., Khalaf A.A.M., Aly M.H. (2020). An Enhanced WDM Optical Communication System Using a Cascaded Fiber Bragg Grating. Opt Quant. Electron.

[6] Talam D.B., El-Badawy E.-S.A., Shalaby H.M.H., Aly M.H. (2020). EDFA Gain Flattening Using Fiber Bragg Gratings Employing Different Host Materials. Opt Quant. Electron.

[7] Sahota J.K., Gupta N., Dhawan D. (2020). Fiber Bragg Grating Sensors for Monitoring of Physical Parameters: A Comprehensive Review. Opt. Express.

[8] Pevec S., Donlagić D. (2019). Multiparameter Fiber-Optic Sensors: A Review. Opt. Express.

[9] Bhaskar C.V.N., Pal S., Pattnaik P.K. (2021). Recent Advancements in Fiber Bragg Gratings Based Temperature and Strain Measurement. Res. Opt..

[10] Herrero A.Á., Fernández-Medina A., Cebollero M., Garranzo-García D., Núñez A., Gonzalo A., Sánchez A., Villanueva J., Parejo P.G., Campos-Jara A. (2022). TuMag for SUNRISE III Mission: Development of the Optical Unit of an Imaging Spectropolarimeter. Proceedings of the Ground-Based and Airborne Instrumentation for Astronomy IX.

[11] Sunrise: Ein Ballongetragenes Sonnenobservatorium. https://www.mps.mpg.de/solar-physics/sunrise.

[12] Rajini-Kumar R., Suesser M., Narayankhedkar K.G., Krieg G., Atrey M.D. (2008). Performance Evaluation of Metal-Coated Fiber Bragg Grating Sensors for Sensing Cryogenic Temperature. Cryogenics.

[13] Merberg G.N., Harrington J.A. (1993). Optical and Mechanical Properties of Single-Crystal Sapphire Optical Fibers. Appl. Opt..

[14] Cheng R., Xia L., Ran Y., Rohollahnejad J., Zhou J., Wen Y. (2015). Interrogation of Ultrashort Bragg Grating Sensors Using Shifted Optical Gaussian Filters. IEEE Photon. Technol. Lett..

[15] Zhu C., Gerald R.E., Huang J. (2020). Progress Toward Sapphire Optical Fiber Sensors for High-Temperature Applications. IEEE Trans. Instrum. Meas..

[16] Arao Y., Koyanagi J., Utsunomiya S., Takeda S., Kawada H. (2009). Analysis of Time-Dependent Deformation of a CFRP Mirror under Hot and Humid Conditions. Mech. Time Depend. Mater..

[17] Catanzaro B.E., Hylton J.C., Bass P., Stumm B.D. (2002). Stability Testing of a Carbon Fiber Composite Optical Bench for Use in the Spacebased LIDAR Mission: CALIPSO (Picasso-Cena). Proceedings of the Earth Observing Systems VII.

[18] Meltz G., Morey W.W., Glenn W.H. (1989). Formation of Bragg Gratings in Optical Fibers by a Transverse Holographic Method. Opt. Lett..

[19] Hill K.O., Meltz G. (1997). Fiber Bragg Grating Technology Fundamentals and Overview. J. Light. Technol..

[20] Marshall G.D., Williams R.J., Jovanovic N., Steel M.J., Withford M.J. (2010). Point-by-Point Written Fiber-Bragg Gratings and Their Application in Complex Grating Designs. Opt. Express.

[21] Othonos A., Kalli K. (1999). Fiber Bragg Gratings: Fundamentals and Applications in Telecommunications and Sensing.

[22] Steenkiste R.J.V. (1996). Strain and Temperature Measurement with Fiber Optic Sensors.

[23] Trutzel M.N., Wauer K., Betz D., Staudigel L., Krumpholz O., Muehlmann H.-C., Muellert T., Gleine W. (2000). Smart Sensing of Aviation Structures with Fiber Optic Bragg Grating Sensors. Proceedings of the Smart Structures and Materials 2000: Sensory Phenomena and Measurement Instrumentation for Smart Structures and Materials.

[24] Frövel M., Gutiérrez C., González S., Carrión G., Cabrerizo F., Pintado J.M. Influence of Temperature and Humidity on the Performance of FBG-Strain Sensors Embedded in CFRP Composites. Proceedings of the 4th European Workshop on Structural Health Monitoring.

[25] Fróvel M., Fernández-Medina Maeso A.B., Mora J., Agüero A., Sor Mendi S., López Heredero R., García-Magariño García A., González Del Val M. (2020). System and Method for Detecting Ice Formation on a Body. U.S. Patent.

[26] Gao H., Jiang Y., Cui Y., Zhang L., Jia J., Jiang L. (2018). Investigation on the Thermo-Optic Coefficient of Silica Fiber Within a Wide Temperature Range. J. Lightwave Technol..

